# Garlic-Derived S-Allylmercaptocysteine Ameliorates Nonalcoholic Fatty Liver Disease in a Rat Model through Inhibition of Apoptosis and Enhancing Autophagy

**DOI:** 10.1155/2013/642920

**Published:** 2013-06-19

**Authors:** Jia Xiao, Rui Guo, Man-Lung Fung, Emily C. Liong, Raymond Chuen Chung Chang, Yick-Pang Ching, George L. Tipoe

**Affiliations:** ^1^Center for Gene and Cell Engineering, Shenzhen Institute of Advanced Technology, Chinese Academy of Sciences, Shenzhen 518055, China; ^2^Department of Anatomy, Li Ka Shing Faculty of Medicine, The University of Hong Kong, Hong Kong; ^3^Department of Physiology, Li Ka Shing Faculty of Medicine, The University of Hong Kong, Hong Kong

## Abstract

Our previous study demonstrated that administration of garlic-derived antioxidant S-allylmercaptocysteine (SAMC) ameliorated hepatic injury in a nonalcoholic fatty liver disease (NAFLD) rat model. Our present study aimed to investigate the mechanism of SAMC on NAFLD-induced hepatic apoptosis and autophagy. Adult female rats were fed with a high-fat diet for 8 weeks to develop NAFLD with or without intraperitoneal injection of 200 mg/kg SAMC for three times per week. During NAFLD development, increased apoptotic cells and caspase-3 activation were observed in the liver. Increased apoptosis was modulated through both intrinsic and extrinsic apoptotic pathways. NAFLD treatment also enhanced the expression of key autophagic markers in the liver with reduced activity of LKB1/AMPK and PI3K/Akt pathways. Increased expression of proapoptotic regulator p53 and decreased activity of antiautophagic regulator mTOR were also observed. Administration of SAMC reduced the number of apoptotic cells through downregulation of both intrinsic and extrinsic apoptotic mechanisms. SAMC also counteracted the effects of NAFLD on LKB1/AMPK and PI3K/Akt pathways. Treatment with SAMC further enhanced hepatic autophagy by regulating autophagic markers and mTOR activity. In conclusion, administration of SAMC during NAFLD development in rats protects the liver from chronic injury by reducing apoptosis and enhancing autophagy.

## 1. Introduction

Nonalcoholic fatty liver disease (NAFLD) is one of the most common chronic liver diseases in Western countries. It ranges from simple fatty liver (steatosis) to nonalcoholic steatohepatitis (NASH) and even cirrhosis [1]. At present, the pathogenesis of NAFLD is not fully understood. Key events that contribute to the initiation and progression of NAFLD are summarized in a “multi-hit” model [2, 3]. In this model, dysregulated metabolism of free fatty acids (FFAs) is considered as the “first-hit” of NAFLD pathogenesis, which leads to insulin resistance and fat accumulation in the liver. Inflammatory response, oxidative stress, apoptosis, and even autophagy serve as “following-hits” that contribute to the ongoing inflammation (NASH). Emerging data suggest that apoptosis plays a critical role in NAFLD-induced liver injury and in the progression from steatosis to NASH and cirrhosis [4–6]. Moreover, the degree of apoptosis is closely associated with the severity of NASH and the stage of fibrosis [7]. Thus, inhibition of apoptosis in the liver may be a useful treatment strategy of NAFLD. 

There are two major apoptotic pathways: intrinsic (mitochondrial) and extrinsic (death receptor) pathways. Both pathways are involved in the pathogenesis of NAFLD [8]. p53 is a transcription factor that controls the activation of both intrinsic and extrinsic apoptotic pathways in response to a variety of stimuli including direct DNA damage, oncogenes, hypoxia, and survival factor deprivation [9]. For intrinsic pathway, p53 enhances the expression of proapoptotic genes, such as Bak1 and Bax, to facilitate the mitochondria-mediated apoptosis. For extrinsic pathway, apart from the signal transduction of death receptors (e.g., Fas and FADD) on the cell membrane, p53 also activates caspase-8 in the cytosol to promote the caspase signaling cascade [9, 10]. Other members of the Bcl-2 family, such as Bcl-2 and Bcl-XL, antagonize the proapoptotic effects mediated by p53 to act as an antiapoptosis mechanism [11]. However, the relationship between the initiation of NAFLD and apoptosis is still poorly understood.

Macroautophagy (hereafter referred to as autophagy) refers to a process where cytoplasmic materials are sequestered and degraded by lysosomal pathway. As a terminal target of insulin signaling, mTOR negatively controls the activity of ULK1 complex and then regulates the autophagic sequestration via vps34 and beclin1. After that, autophagosomes fuse with lysosome to degrade target cytosolic contents through the action of Atg 5, 12, and LC3 [12]. In the liver, autophagy is believed to exert several important physiological functions, including starvation adaptation, quality control (to prevent the accumulation of degenerating proteins and organelles), and prevention of tumorigenesis [13]. However, the exact role of autophagy during NAFLD progression remains largely unknown.

S-allylmercaptocysteine (SAMC) is a water-soluble compound of aged garlic. It is a major *in vivo* metabolic product of diallyl disulfide and allicin, the organo-sulfur compounds of raw garlic [14]. SAMC has been characterized for its anticancer property both *in vivo* and *in vitro* [15–17]. In addition, SAMC also plays a preventive role in an acetaminophen-induced acute liver injury model through the inhibition of the activity of cytochrome P450 2E1 (CYP2E1) [18]. We previously demonstrated the protective properties of SAMC in both carbon tetrachloride-induced acute liver injury model [19] and NAFLD-induced chronic liver injury model [20]. In these studies, SAMC reduces the key events that contribute to the hepatic damage including oxidative stress, inflammation, and necrosis. However, whether the application of SAMC could alleviate apoptosis in NAFLD liver injury is still largely unknown. In the current study, we investigated the antiapoptotic and autophagic enhancing effects of SAMC in a NAFLD rat model. Signaling pathways regulated by SAMC on hepatic apoptosis and autophagy have also been characterized.

## 2. Materials and Methods

### 2.1. Reagents

SAMC pure powder was kindly given by Dr. Patrick M. T. Ling (Queensland University of Technology, Australia) and originally from Wakunaga Co. Ltd (Osaka, Japan). The purity of the SAMC powder is more than 95% by HPLC analysis. It does not contain any other garlic compound such as SAC or allicin. SAMC was dissolved in a phosphate buffered saline containing 10% L-dextrose and 1% gum Arabic (w/v) at pH 4.5. Antibodies against Bcl-2, Bcl-XL, Bak1, Bax, vps34, and phosphorylated phosphoinositide 3-kinase (PI3 K) p85*α* at Tyr508 were purchased from Santa Cruz Biotechnology (Santa Cruz, CA, USA). Antibodies of phosphorylated liver kinase B1 (LKB1) at Ser428, total LKB1, phosphorylated AMP-activated protein kinase (AMPK) at Thr172, total AMPK, phosphorylated p53 at Ser15, total p53, phosphorylated Akt at Ser473, total Akt, total PI3 K (p85 subunit), cytochrome c, TNF-related apoptosis-inducing ligand (TRAIL), Fas, Fas-associated protein with death domain (FADD), cleaved caspase-3, cleaved caspase-8, phosphorylated mTOR at Ser2448, mTOR, beclin 1, Atg12, LC3 II, and p62 were from Cell Signaling Technology (Danvers, MA, USA).

### 2.2. Animals and Treatments

Eight weeks healthy female SD rats with body weight ranging from 180–200 g were purchased from the Laboratory Animal Unit (LAU), The University of Hong Kong. Rats were kept under standard conditions for three days before starting of the experiment with free access to animal chow and tap water. The animals were divided into four groups (*n* = 7 in each group), namely, (1) control group; (2) NAFLD group; (3) SAMC treatment only group (200 mg/kg in solvent, intraperitoneal injection, three times per week); and (4) NAFLD and SAMC cotreatment group. Pilot studies on hepatic histology and serum ALT showed that this solvent had no hepatic toxicity. The development of NAFLD in rats, including the recipe and preparation protocols of diet, was performed based on our previously described voluntary oral feeding NAFLD animal model [18]. The optimum dosage of SAMC was previously shown to be effective in protecting the liver from both acute and chronic injury [19, 20]. Instead of oral administration in a dietary supplement form, SAMC was intraperitoneally injected to avoid possible degradation prior to absorption through the gastrointestinal tract (GIT). After eight weeks, the rats were euthanized by an overdose of anesthesia according to the protocols approved by the Committee on the Use of Live Animals in Teaching and Research at The University of Hong Kong. The Laboratory Animal Unit of the University of Hong Kong is fully accredited by the Association for Assessment and Accreditation of Laboratory Animal Care International (AAALAC international). Liver samples were collected for further analysis.

### 2.3. Processing of Tissue and TUNEL Assay

Liver tissue samples were fixed in 10% phosphate-buffered formalin processed for histology and embedded in paraffin blocks. Five-micrometer tissue sections were subjected to hematoxylin and eosin (H&E) staining and terminal deoxynucleotidyl transferase-mediated dUTP nick-end labeling (TUNEL) assay using an *in situ *cell death detection kit (Roche Diagnostics, Basel, Switzerland). After H&E staining, hepatic injury was evaluated by using the NAFLD activity score (NAS) system as previously described [20]. For TUNEL assay, TUNEL-positive parenchymal and nonparenchymal cell signals were quantified in terms of the intensity of the red stain. This parameter is represented by the mean optical density in ten random fields per section per animal using the ImageJ software (NIH, Bethesda, MD, USA).

### 2.4. Western Blot Analysis

Cytosolic protein of each liver sample was extracted by using NE-PER protein extraction system (Pierce Biotechnology, Rockford, IL, USA) with the addition of Halt phosphatase inhibitor cocktail (Pierce). Before Western blot, protein was diluted and mixed with 2 × sample buffer (0.1 M Tris-HCl, pH 6.8, 20% glycerol, 4% sodium dodecyl sulfate, 0.2% Bromophenol Blue, 5.25%  *β*-mercaptoethanol). The mixture was denatured at 99°C for 5 min and followed by electrophoresis in a 10% polyacrylamide gel. The protein was then transferred to an Immun-Blot PVDF Membrane (Bio-Rad) in a TE series transfer electrophoresis unit (Hoefer Inc., Holliston, MA, USA). The membrane was then incubated in blocking buffer (5% nonfat milk powder in TBST, 100 mM Tris-HCl, pH 7.5, 0.9% NaCl, 0.1% Tween 20) for 1 hour followed by incubation with appropriate primary antibodies in TBST overnight at 4°C with gentle agitation. On the following day, the membrane was washed with TBST and incubated with appropriate secondary antibodies for 2 h at room temperature. Beta-actin was used as the internal control. After washing off the unbound antibody with TBST, the expression of the antibody-linked protein was determined by an ECL Western Blotting Detection Reagents (GE Healthcare). The optical density of the bands was measured and quantified by ImageJ software (National Institute of Health, MD). The ratio of the optical density of the protein product to the internal control was calculated and was expressed as a percentage of the control expression by ImageJ.

### 2.5. Statistical Analysis

Data from each group were expressed as means ± SEM. Statistical comparison between groups was done using the Kruskal-Wallis test followed by Dunn's post hoc test to compare all groups. A *P* < 0.05 was considered to be statistically significant (Prism 5.0, Graphpad software, Inc., San Diego, CA, USA).

## 3. Results

### 3.1. SAMC Cotreatment Improved Hepatic Histology during NAFLD Development

**Figure 1:**
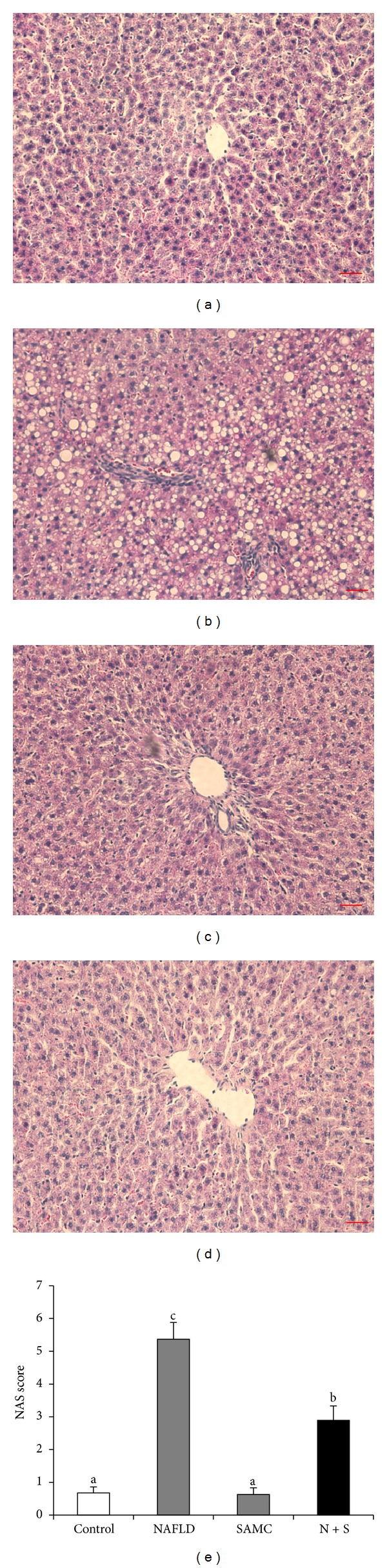
Cotreatment with SAMC during NAFLD development improved hepatic histology in rats. ((a)–(d)) Representative images of H&E staining in the rat liver sections ((a) control, (b) NAFLD, (c) SAMC, (d) NAFLD + SAMC) and (e) quantitative data of NAS score of H&E staining. Data presented are expressed as Mean ± SEM (*n* = 7) and experimental groups marked by different letters represented significant differences between groups at *P* < 0.05 (Kruskal-Wallis test followed by Dunn's post hoc test). Magnification: 200x. Bar: 20 microns. N + S: NAFLD + SAMC cotreatment.

Eight-week induction of NAFLD by high-fat diet induced showed increase in lipid accumulation and inflammatory foci deposition in the rat liver. SAMC cotreatment significantly improved the hepatic histology by reducing the fatty droplets and inflammatory foci number without influencing the healthy rats (Figures 1(a)–1(d)). NAS quantification of liver sections further confirmed the beneficial effects of SAMC cotreatment on hepatic histology (Figure 1(e)).

### 3.2. Addition of SAMC Reduced Apoptosis in the Liver during NAFLD Development

**Figure 2:**
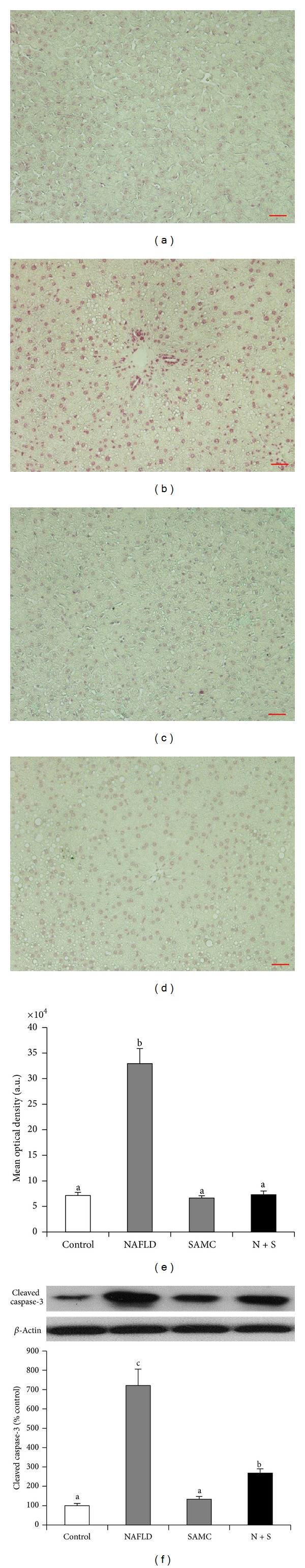
Addition of SAMC during the development of NAFLD reduced the hepatic apoptosis in rats. ((a)–(d)) Representative results of TUNEL assay in the rat liver sections by fast red staining ((a) control, (b) NAFLD, (c) SAMC, (d) NAFLD+SAMC) and (e) quantitative data of TUNEL assay results (a.u. = arbitrary unit). (f) Protein expression of cleaved (activated) caspase-3 was measured by Western blot and then quantified by ImageJ software. Data presented are expressed as mean ± SEM (*n* = 7), and experimental groups marked by different letters represented significant differences between groups at *P* < 0.05 (Kruskal-Wallis test followed by Dunn's post hoc test). Magnification: 200x. Bar: 20 microns. N + S: NAFLD + SAMC cotreatment.

After 8 weeks of NAFLD induction using high-fat diet, hepatic apoptosis in NAFLD rats was more evident than that in other three groups (~3.5-fold), as shown by the quantification of TUNEL assay staining (Figure 2(e)). Cotreatment with 200 mg/kg SAMC significantly reduced the intensity of hepatic apoptotic positive signal comparable to the control level in the liver section (Figures 2(a)–2(d)). Vehicle-treated SAMC group rats did not show increase in the intensity of apoptotic signals when compared with the control group (Figure 2(c)). As the central apoptotic signaling pathway, caspase-3 is activated under the signals from both intrinsic and extrinsic apoptotic pathways [21]. In NAFLD rats, the expression level of cleaved (activated) caspase-3 was markedly higher than the control level (~7.2-fold), which was consistent with the TUNEL assay. Addition of SAMC significantly and markedly reduced the level of the activated caspase-3 induced by a high-fat diet (Figure 2(f)).

### 3.3. Intrinsic Apoptotic Signaling Pathway Components Involved in SAMC Attenuation

**Figure 3:**
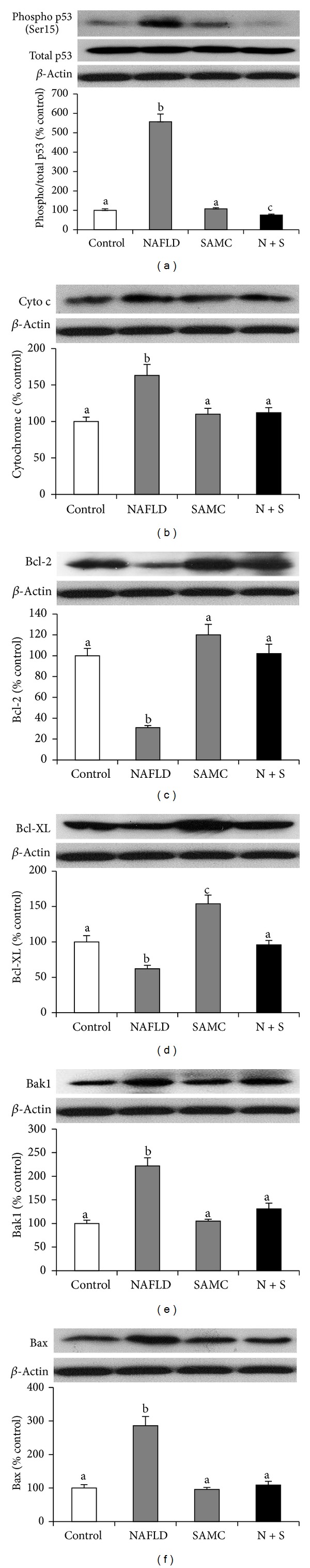
Addition of SAMC attenuated intrinsic apoptotic pathway components through p53 during the development of NAFLD. Protein expressions of (a) phosphorylated and total p53, (b) cytochrome c, (c) Bcl-2, (d) Bcl-XL, (e) Bak1, and (f) Bax were measured by Western blot and then quantified by ImageJ software. Data presented are expressed as mean ± SEM (*n* = 7), and experimental groups marked by different letters represented significant differences between groups at *P* < 0.05 (Kruskal-Wallis test followed by Dunn's post hoc test). N + S: NAFLD + SAMC cotreatment.

In NAFLD rats, the protein level of phosphorylated p53 was highly elevated, indicating an activation of the master regulator of cellular apoptosis. Cotreatment with SAMC during NAFLD development significantly reduced the phosphorylated p53 expression to the control level without significantly disturbing its baseline and the total form of p53 expressions (Figure 3(a)). As an important intrinsic intermediate in apoptosis, the protein level of cytochrome c was also upregulated in NAFLD rats but attenuated in SAMC cotreatment rats (Figure 3(b)). The antiapoptotic members of the Bcl-2 family (Bcl-2 and Bcl-XL) showed inhibited expression during the development of NAFLD, while the level of proapoptotic members (Bak1 and Bax) was upregulated (Figures 3(c)–3(f)). Administration of SAMC potently counter-acted the effects of NAFLD on these Bcl-2 family members through the intrinsic apoptotic pathway.

### 3.4. Extrinsic Apoptotic Signaling Pathways Components Involved in SAMC Attenuation

**Figure 4:**
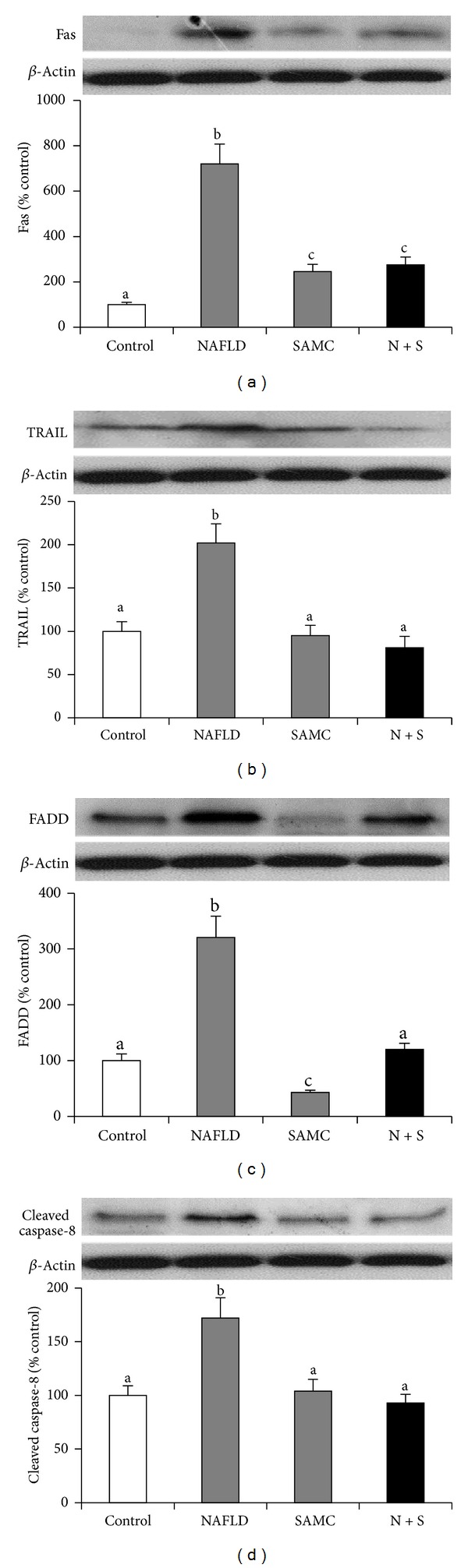
Addition of SAMC attenuated extrinsic apoptotic pathway components during the development of NAFLD. Protein expression of (a) Fas, (b) TRAIL, (c) FADD, and (d) cleaved caspase-8 were measured by Western blot and then quantified by ImageJ software. Data presented are expressed as mean ± SEM (*n* = 7), and experimental groups marked by different letters represented significant differences between groups at *P* < 0.05 (Kruskal-Wallis test followed by Dunn's post hoc test). N + S: NAFLD + SAMC cotreatment.

To further examine the effects of NAFLD and SAMC on the extrinsic apoptotic pathway, protein expressions of key extrinsic apoptotic pathway components, including Fas, TRAIL, FADD, and cleaved caspase-8, were measured by Western blot. The expression level of Fas, TRAIL, FADD, and cleaved caspase-8 was upregulated during the NAFLD progression by 7.1-fold, 2.0-fold, 3.2-fold, and 1.7-fold, respectively. Administration of SAMC significantly reduced the elevated expressions of these proteins comparable to the control levels (Figures 4(a)–4(d)). SAMC treatment alone did not influence the basal expression of TRAIL and cleaved caspase-8 but increased basal Fas level and decreased basal FADD level.

### 3.5. SAMC Alleviated Hepatic Apoptosis through Targeting LKB1/AMPK and PI3 K/Akt Pathways

**Figure 5:**
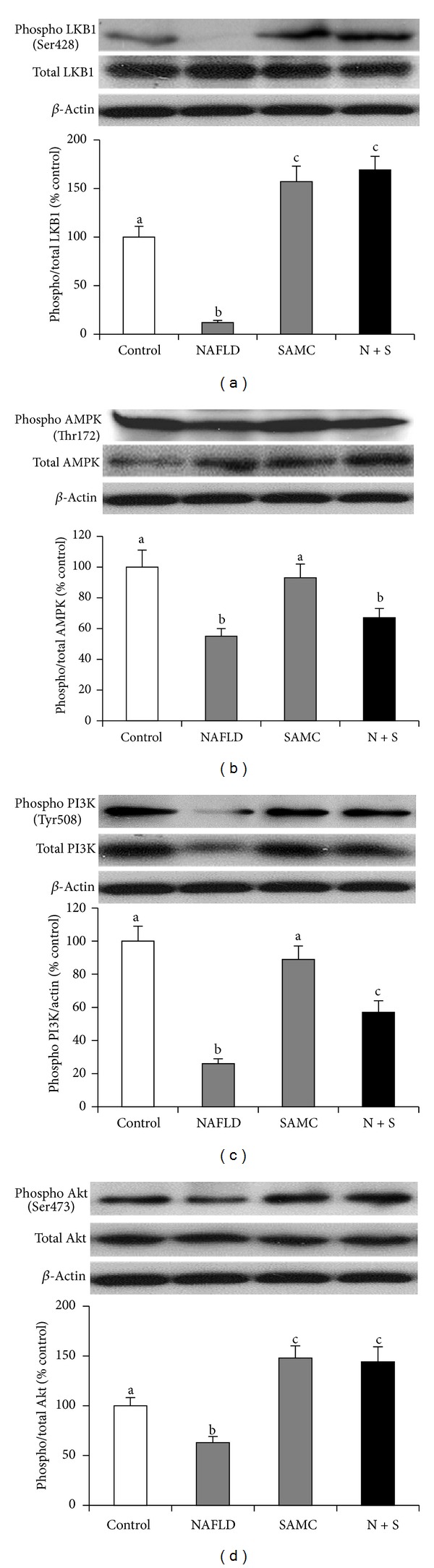
Addition of SAMC reduced NAFLD-induced hepatic apoptosis via modulating the LKB1/AMPK and PI3 K/Akt pathways. Protein expressions of phosphorylated and total (a) LKB1, (b) AMPK, (c) PI3 K, and (d) Akt were measured by Western blot and then quantified by ImageJ software. Data presented are expressed as mean ± SEM (*n* = 7), and experimental groups marked by different letters represented significant differences between groups at *P* < 0.05 (Kruskal-Wallis test followed by Dunn's post hoc test). N + S: NAFLD + SAMC cotreatment.

To explore the signaling pathways involved in SAMC attenuated apoptosis, we measured the phosphorylation and total forms of key components from two kinase pathways, namely, LKB1/AMPK and PI3 K/Akt signaling pathways. Development of NAFLD in rats inhibited the phosphorylation of LKB1, AMPK, PI3 K, and Akt proteins (Figures 5(a)–5(d)). The influence of NAFLD on total LKB1, AMPK, and Akt was not obvious, while total PI3 K expression was inhibited by NAFLD. Addition of SAMC dramatically restored the phosphorylation form of LKB1 and Akt to levels that were higher than control (Figures 5(a) and 5(d)). Treatment of SAMC also upregulated the phosphorylated and total protein expressions of PI3 K when compared with the NAFLD group (Figure 5(c)). For phosphorylated AMPK, SAMC also slightly restored its level when compared with the NAFLD rat level, although the change was not statistically significant (Figure 5(b)). 

### 3.6. SAMC Treatment Further Enhanced Autophagy through Inhibition of mTOR Activity

**Figure 6:**
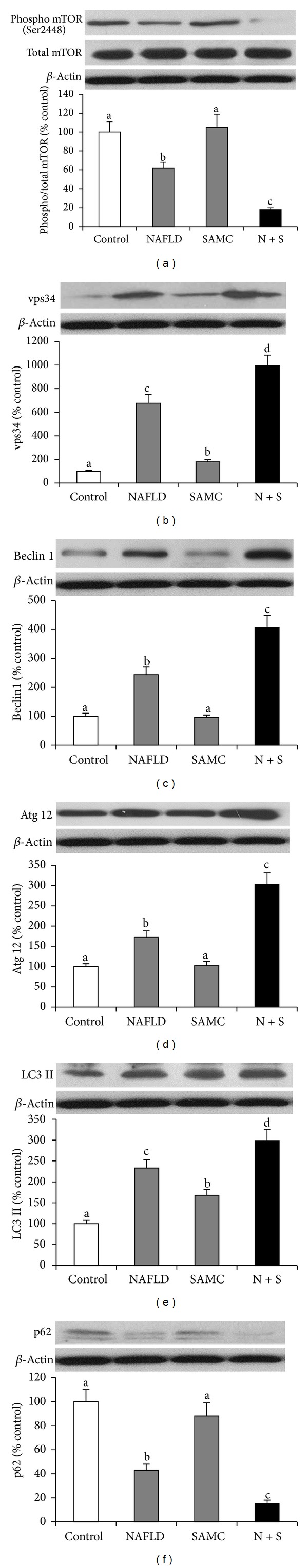
Cotreatment with SAMC further enhanced hepatic autophagy through inhibition of mTOR activity. Protein expressions of (a) phosphorylated and total mTOR, (b) vps34, (c) beclin 1, (d) Atg 12, (e) LC3 II and (f) p62 were measured by Western blot and then quantified by ImageJ software. Data presented are expressed as mean ± SEM (*n* = 7), and experimental groups marked by different letters represented significant differences between groups at *P* < 0.05 (Kruskal-Wallis test followed by Dunn's post hoc test). N + S: NAFLD + SAMC cotreatment.

NAFLD rats showed increased expression level of autophagic markers during NAFLD progression, including vps34, beclin 1, Atg 12, and LC3 II, with inhibited phosphorylation level of autophagic inhibitor mTOR (Figures 6(a)–6(e)). Interestingly, cotreatment with SAMC further enhanced the expression level of vps34, beclin 1, Atg 12, and LC3 II. It also further decreased the phosphorylation of mTOR, indicating a further induction of hepatic autophagy after NAFLD progression through inhibition of mTOR activity (Figures 6(a)–6(e)). As an ubiquitin binding protein for autophagy, the protein expression of p62 was downregulated in the NAFLD group and further reduced by the cotreatment of SAMC (Figure 6(f)).

## 4. Discussion

Despite the huge effort put in the prevention and treatment of NAFLD from researchers and clinicians, there are few options to retard or even reverse the progression of this disease. As to date, weight loss is the most recognized therapeutic method to improve liver injury induced by NAFLD [22]. Recently, several drugs have been assessed for the treatment of NAFLD, including antiobesity regimens, insulin sensitizers, antihyperlipidemics, and antioxidants. However, a few of them showed very positive outcomes [23]. We have reported that administration of 200 mg/kg SAMC during the development of NAFLD in a rat model could attenuate the histolopathogical changes, lipid metabolism dysfunction, oxidative stress, and inflammation through kinase- and transcription-factor-dependent pathways with minimal side effects on healthy animals [20]. In the current study, we demonstrated the antiapoptotic and proautophagic properties of SAMC cotreatment. During NAFLD development, both intrinsic and extrinsic apoptotic pathways have been activated to transduce death signals to the functional protein caspase-3 under the actions of p53. As the upstream regulating pathways, both LKB1/AMPK and PI3 K/Akt pathways were inhibited to further facilitate the process of apoptosis. Addition of SAMC targeted both intrinsic and extrinsic pathways through restoring the LKB1/AMPK and PI3 K/Akt pathways, leading to reduced caspase-3 activity and apoptosis in the liver. In addition, treatment of SAMC further enhanced the hepatic autophagy through the inhibition of mTOR, contributing to the ameliorative effects of SAMC.

Apoptosis of liver cells and adipocytes is often found in NAFLD patients and experimental animals [4, 24]. It is considered as a critical factor for the progression of NAFLD to NASH [8]. Inhibition of excessive apoptosis in the liver may be helpful in the treatment of NASH experimentally and clinically. In response to cellular damage, such as hypoxia, DNA damage, and fat accumulation, the p53 tumor suppressor is activated to inhibit cell proliferation through promotion of intrinsic and extrinsic apoptotic pathways [9]. Previous study found that the extrinsic pathway of apoptosis (especially the activation of Fas/FasL system) may be a central event for the induction of apoptosis in NAFLD [7, 25]. Another report also showed that the activation of p53 and TRAIL receptor expression is associated with apoptosis in a methionine and choline deficient (MCD) diet model [26]. Therefore, it is very clear that both intrinsic and extrinsic pathways of apoptosis are activated in NAFLD despite the action of p53, which is consistent with our current findings of clinically relevant and not genetically modified NAFLD rat model [27]. Interestingly, several previous reports demonstrated that in cancer cell, addition of SAMC induced apoptosis by microtubule depolymerization, JNK1, and caspase-3 activation [17, 28]. The discrepancy of results between these reports and our current study may be due to different microenvironment. In cancer cells, apoptosis is a beneficial event which can retard the proliferation of tumor cells, whereas in NAFLD rats, apoptosis is a detrimental event responsible for the progression and severity of NAFLD. Therefore, SAMC may exert distinct action on apoptosis under different circumstances, whichever is more beneficial to the host. Indeed, the underlying mechanisms for this interesting phenomenon require further investigations.

The detailed function and mechanism of autophagy in NAFLD development are not fully elucidated. Recent studies pointed out that autophagy may selectively target lipid droplets within hepatocytes for degradation, leading to reduction of steatosis. This process is called lipophagy [29]. Pharmacological inhibition of vps34 by 3-methyladenine (3MA) increases the triglyceride (TG) contents in normal cell or cell treated with unsaturated fatty acid. Inhibition of negative regulator of autophagy, mTOR, by rapamycin decreases oleic acid-induced TG levels in cultured hepatocytes [29, 30] and fatty liver mouse model [31]. Therefore, enhancing autophagy is considered as a novel therapeutic strategy for NAFLD therapy [32]. In this study, SAMC enhanced the hepatic autophagy during NAFLD development, with further reduced activity of mTOR, indicating a mTOR-directed pathway. Whether this process is directly related to the reduction of lipid contents in hepatocytes needs future investigations.

To further investigate the upstream signaling regulators of apoptosis and autophagy in the liver, we assessed the phosphorylation and total forms of LKB1/AMPK and PI3 K/Akt pathways and found that reactivation of these two pathways contributed to the cell survival during NAFLD. AMPK is an important enzyme response to energy deprivation and, in some cases, cellular stress to induce apoptosis through the AMPK-p53 axis [33]. During NAFLD, AMPK increases the transport of FFAs into the mitochondria, as well as promotes *β*-oxidation, thus restoring energy balance [34]. In many cases, activation of AMPK protected cells from apoptosis. In an iron-induced hepatic oxidative stress and liver injury model, addition of sauchinone, a bioactive lignan, activated the LKB1/AMPK pathway, resulting in inhibition of apoptosis in the liver [35]. Moreover, an *in vitro* study using HepG2 cell line found that resveratrol attenuates arachidonic acid and iron-induced apoptosis through activation of LKB1/AMPK pathway [36]. Recent study found that activation of hypothalamic autophagy PI3 K/Akt pathway has also been found to play an important role in the impairment of mitochondria during NAFLD development. In a high-fat diet fed NAFLD rat model, reduced phosphorylated form of PI3 K and Akt and total form of PI3 K were observed with hepatic apoptosis. Treatment of pharmacological inhibitors of PI3 K or Akt instead of high-fat diet mimicked such phenomena [37]. Thus, in this study, the modulation of the activity of LKB1/AMPK and PI3K/Akt pathways by SAMC administration may partly be involved in its antiapoptotic effect during NAFLD development. However, further investigations are needed to clarify the interactions between these pathways and p53, as well as the possible involvement of other apoptosis-related signaling pathways. In addition, it is not clear whether the antiapoptotic effects of SAMC on NAFLD are a direct effect or a consequence of “upstream” antioxidant and anti-inflammatory effects. Although some studies proposed the regulatory roles of LKB1/AMPK and PI3 K/Akt pathways in autophagy [38], in this study, it is suggested that the further enhancement of autophagy by SAMC cotreatment was not through these two pathways. Detailed mechanisms for distinct regulation of apoptosis and autophagy by SAMC are waiting for further study.

In conclusion, our results clearly showed the antiapoptotic and proautophagy properties of SAMC during the development of NAFLD in a rat model. The protective effect of SAMC was partly through modulating both p53-dependent intrinsic and extrinsic apoptotic pathways, as well as the inhibition of mTOR activity. Restoration of LKB1/AMPK and PI3 K/Akt pathways also contributed to this protective effect of SAMC.
